# Nutritional Regulation of Gene Expression: Carbohydrate-, Fat- and Amino Acid-Dependent Modulation of Transcriptional Activity

**DOI:** 10.3390/ijms20061386

**Published:** 2019-03-19

**Authors:** Diego Haro, Pedro F. Marrero, Joana Relat

**Affiliations:** 1 Department of Nutrition, Food Sciences and Gastronomy, School of Pharmacy and Food Sciences, Food Campus Torribera, University of Barcelona, E-08921 Santa Coloma de Gramenet, Spain; dharo@ub.edu (D.H.); pedromarrero@ub.edu (P.F.M.); 2 Institute of Biomedicine of the University of Barcelona (IBUB), CIBER Physiopathology of Obesity and Nutrition (CIBER-OBN), Instituto de Salud Carlos III, E-28029 Madrid, Spain

**Keywords:** carbohydrates, amino acids, fatty acids, carbohydrate-responsive element binding protein, peroxisome proliferator-activated receptors, amino acid response, activating transcription factor 4, TORC1 signaling

## Abstract

The ability to detect changes in nutrient levels and generate an adequate response to these changes is essential for the proper functioning of living organisms. Adaptation to the high degree of variability in nutrient intake requires precise control of metabolic pathways. Mammals have developed different mechanisms to detect the abundance of nutrients such as sugars, lipids and amino acids and provide an integrated response. These mechanisms include the control of gene expression (from transcription to translation). This review reports the main molecular mechanisms that connect nutrients’ levels, gene expression and metabolism in health. The manuscript is focused on sugars’ signaling through the carbohydrate-responsive element binding protein (ChREBP), the role of peroxisome proliferator-activated receptors (PPARs) in the response to fat and GCN2/activating transcription factor 4 (ATF4) and mTORC1 pathways that sense amino acid concentrations. Frequently, alterations in these pathways underlie the onset of several metabolic pathologies such as obesity, insulin resistance, type 2 diabetes, cardiovascular diseases or cancer. In this context, the complete understanding of these mechanisms may improve our knowledge of metabolic diseases and may offer new therapeutic approaches based on nutritional interventions and individual genetic makeup.

## 1. Introduction

The discovery of the galactose operon in bacteria represented a key finding for the study of the regulation of metabolism. That work showed how, by modifying the level of expression of specific enzymes, bacteria can adapt their metabolism to meet their nutritional needs, and it connected, for the first time, changes in enzymatic activity to the transcriptional control of gene expression [1]. It is now commonly accepted that transcriptional regulation also contributes to metabolic homeostasis in complex organisms.

The alteration of the mechanisms controlling gene expression (from transcription to translation), may lead to the development of metabolic diseases. Thus, understanding the effect of nutrients on gene expression may improve our knowledge of metabolic diseases and may offer new therapeutic approaches based on nutritional interventions and individual genetic makeup. For instance, the risk of having a metabolic syndrome (MetS) caused by a disruption of energy homeostasis is associated with overweight and obesity. This association stresses the link between lipid and glucose metabolism. While the treatment of dyslipidemia and diabetes characteristic of the metabolic syndrome can be achieved by drugs targeting cholesterol synthesis or pancreatic beta cell function, other metabolic dysfunctions typical of this situation have a more complicated treatment. The family of peroxisome proliferator-activated receptors (PPARs), metabolic sensors involved in the control of lipid and glucose metabolism, is a good example of how knowledge of the mechanisms that control gene expression offer new therapeutic opportunities. In this sense, the thiazolidinediones (TZDs), PPARγ agonists, are used as potent hypoglycemic agents.

**Figure 1:**
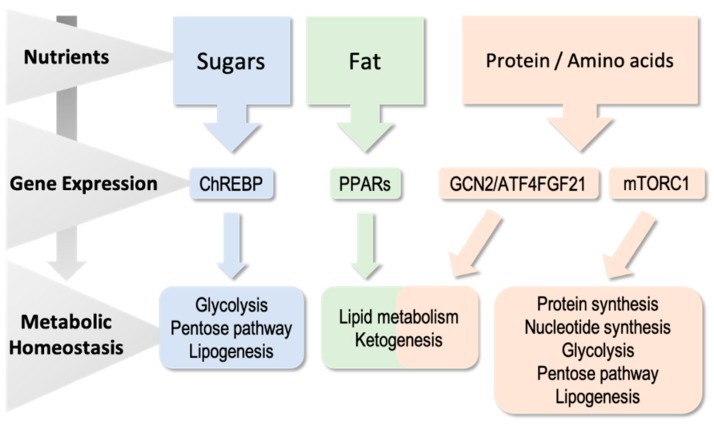
Mammals detect an abundance of nutrients such as sugars, fat and amino acids, and provide a metabolic response most times through the control of gene expression (from transcription to translation). Sugars signaling mainly goes through the carbohydrate-responsive element binding protein (ChREBP). Peroxisome proliferator-activated receptors (PPARs) are the responsible response to fat, and the GCN2/activating transcription factor 4 (ATF4) and mTORC1 pathways sense amino acid concentrations.

The purpose of this review is to highlight current knowledge of how transcriptional control participates in homeostatic energy balance; particularly, how carbohydrates, lipids and amino acids—nutrients that can be used as energy sources—modulate transcriptional activity to achieve metabolic homeostasis (Figure 1). We will not discuss in this review other pathways that are also modulated by nutrients, such as the complex regulatory framework responsible for cholesterol homeostasis that includes the sterol regulatory element binding proteins (SREBPs), nor will we discuss members of the nuclear receptor family of metabolic sensors, such as the oxysterol-activated receptors, liver X receptors (LXRs) and the bile acid-activated farnesoid X receptor (FXR). We will not comment, either, on the important impact of nutrients on the epigenetic mechanisms of gene regulation.

## 2. Sugar. The Carbohydrate-Responsive Element Binding Protein (ChREBP)

Metabolic homeostasis and energy balance require a precise control of glucose and lipid metabolism. Hormonal regulation in response to glucose availability is mainly responsible for this control, but in this review we will refer exclusively to the mechanisms that explain a direct effect of different metabolites on the transcription of genes that code for enzymes involved in metabolic homeostasis.

The regulation of the metabolic pathways involved in glucose homeostasis is carried out in part by the transcriptional control of the genes coding for the regulatory enzymes of those pathways. Shortly after the elevation of glucose levels in the liver, several key enzymes of glycolysis and lipogenesis are post-translationally activated by well-known mechanisms. A high carbohydrate diet also induces transcription of the genes encoding these enzymes, including glucokinase (GK) [2] and pyruvate kinase [3,4] for glycolysis, ATP citrate lyase [5], acetyl CoA carboxylase [6], fatty acid synthase (FASN) [7] and stearoyl-CoA desaturase 1 (SCD1) [8] for lipogenesis and glucose 6-phosphate dehydrogenase [9] for the pentose pathway, thus promoting the storage of sugars as triglycerides (TGs).

**Figure 2:**
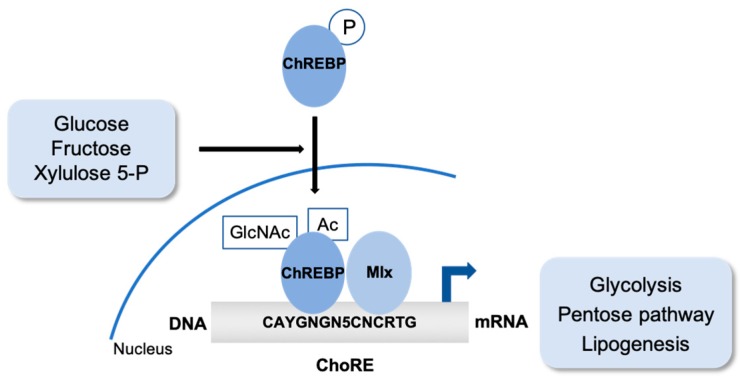
ChREBP is a basic helix–loop–helix leucine zipper transcription factor. In response to glucose and fructose, this protein forms a heterodimer with its partner Mlx and binds and activates the transcription of target genes that contain carbohydrate response element (ChoRE) motifs. This regulation plays a critical role in sugar-induced lipogenesis and global glucose homeostasis. The mechanisms of ChREBP activation involve several glucose metabolites, pathways and post-translational modifications including phosphorylation, acetylation and O–GlcNAcylation.

The mechanism by which carbohydrates regulate transcription of these genes besides the transcriptional control exercised by insulin and glucagon and their signaling cascade, was finally unraveled by the purification and characterization of the carbohydrate-responsive element binding protein (ChREBP). ChREBP is a basic helix–loop–helix leucine zipper transcription factor encoded by a gene localized in the region of chromosome 7q11.23 that is deleted in patients with Williams–Beuren syndrome, a multisystemic developmental disorder [10]. In response to glucose and fructose, this protein forms a heterodimer with its partner Mlx and binds and activates the transcription of target genes that contain carbohydrate response element (ChoRE) motifs. This regulation plays a critical role in sugar-induced lipogenesis and glucose global homeostasis through the coordination of hepatic intermediary metabolism, carbohydrate digestion and transport [11,12] (Figure 2).

Besides its role as a glucose sensor, ChREBP has also been described as essential for fructose-induced lipogenesis in both the small intestine and liver [12,13]. In fact, an acute and robust ingestion of fructose, but not of glucose, activates hepatic ChREBP. In this context, it has been published that ChREBP contributes to some of the physiological effects of fructose on sweet taste preference and glucose production through regulation of, for instance, fibroblast growth factor-21 (FGF21) or the catalytic subunits of glucose-6-phosphatase. It has been recently demonstrated that ChREBP loss of function is essential for the fructose-dependent increase of plasmatic levels of FGF21, and that under high-fructose diets an absence of FGF21 leads to liver disease. A correlation between circulating FGF21 and rates of de novo lipogenesis has also been shown in humans. Altogether, these results indicate that the signaling axis sugar(fructose)–ChREBP–FGF21 may play a role in liver pathogenesis [14]. Finally, it has been suggested that the restriction of fructose over intake will be beneficial for preventing irritable bowel syndrome modulating the impact of ChREBP activity in fructose metabolism [15].

Two isoforms of ChREBP have been identified. A novel variant called ChREBPβ expressed from an alternative promoter in a glucose- and ChREBPα-dependent manner was identified in adipose tissue [16]. That article suggests a mechanism whereby, through two steps, the glucose-induced ChREBPα transcriptional activity induces the expression of the more potent isoform, ChREBPβ. A negative feedback loop by which glucose-induced ChREBPβ downregulates ChREBPα signaling has been described in pancreatic islets, providing new insight into the physiological role of islet ChREBPβ and the regulation of glucose-induced gene expression [17].

### 2.1. ChREBP Post-Translational Modifications

The mechanisms of ChREBP activation by glucose involve several glucose metabolites, pathway, and post-translational modifications, including phosphorylation, acetylation and O-GlcNAcylation [18,19].

Phosphorylation/dephosphorylation-dependent subcellular localization and activity is a key regulatory mechanism of ChREBP activity in response to glucose level [20,21,22]. ChREBP is regulated by nuclear/cytosol trafficking via interaction with 14-3-3 proteins, CRM-1 or importins [23,24]. A decrease in glucose concentration results in ChREBP phosphorylation by PKA, a complex formation with 14-3-3 and the localization in the cytosol of an inactive pool of ChREBP–14-3-3 complex [24]. The increase in glucose levels raises the concentration of xylulose 5-phosphate (X5P), a pentose shunt intermediate that leads to the activation of a specific protein phosphatase that dephosphorylates ChREBP. The ChREBP dephosphorylation is a necessary event for its nuclear localization and transcriptional activation [24,25]. Elsewhere, other metabolites have been proposed as potential regulators of ChREBP translocation and the role of PP2A activity and X5P as a signaling metabolite in the liver has been challenged [26]. That study reveals that G6P produced by GK, but not X5P, is essential for both ChREBP nuclear translocation and transcriptional activity induced by glucose in liver cells. Fructose-2, 6-P2, the major regulator of glycolysis and gluconeogenesis, has also been implicated in this response [27].

High glucose levels induce ChREBP acetylation and O-GlcNAcylation. These modifications do not influence ChREBP localization, but instead favor the recruitment to its target genes [28,29]. The biological consequences of the site-specific O-GlcNAcylation dynamics of ChREBP have recently been reviewed. Under high-glucose conditions, the phosphorylation of Ser514 increases the ChREBP O-GlcNAcylation and maintains its transcriptional activity. Moreover, Ser839 O-GlcNAcylation is essential for Mlx heterodimerization, DNA-binding and therefore transcriptional activity, but also for ChREBP nuclear export, partially due to stronger interactions with CRM1 and 14-3-3 [30].

O-GlcNAc is a nutrient-sensitive modification notably apt for the integration of several metabolic signals because the hexosamine biosynthetic pathway (HBP) is a central player in nutrient sensing. This is a key pathway for regulating nutrient processing because its final product, UDP-*N*-acetylglucosamine, is synthesized based on nutrient availability, and this activated sugar-nucleotide is utilized to produce a potent post-translational regulatory modification [31]. 

### 2.2. ChREBP Partners to Regulate Gene Expression and Metabolism

ChREBP transcriptional activity depends on the presence of other cofactors and transcriptional factors such as the members of nuclear receptors family hepatic nuclear factor 4 (HNF-4), LXR, FXR or the thyroid hormone receptor (TR) [32,33].

FXR is a key transcription factor of bile acid metabolism that was recently shown to interact directly with ChREBP, acting as a repressor on the ChoRE of glycolytic genes [34]. Interestingly, similarly to ChREBP, FXR is O-GlcNAcylated in response to glucose. It has been described that ChREBP and FXR O-GlcNAcylation can modify their reciprocal affinity and transcriptional activity [35].

An important role for LXR linking hepatic glucose utilization to lipid synthesis has been suggested. LXR α/β double-knockout mice show reduced feeding-induced nuclear O-GlcNAcylated ChREBPα, ChREBPα activity and lipogenic gene expression in the liver. The study of the effects of high-fructose or high-glucose feeding on hepatic gene expression from fasted and fasted–refed wild type and LXRα knockout mice suggests that, in mice, LXRα is an important regulator of hepatic lipogenesis and ChREBPα activity upon glucose, but not fructose intake [36].

A specific cross-talk between ChREBP and PPARα has been shown for the glucose-mediated induction of FGF21 expression. In hepatic PPARα knockout mice, the glucose-dependent induction of FGF21 expression associated with an increased sucrose preference is blunted under a carbohydrate administration. The absence of response is due to diminished ChREBP binding onto FGF21 ChoRE, indicating that PPARα is required for the ChREBP-induced glucose response of FGF21 [37].

ChREBP provides hepatoprotection against a high-fructose diet also by preventing overactivation of cholesterol biosynthesis and the subsequent activation of the proapoptotic arm of the unfolded protein response (UPR). A role has also been identified for ChREBP in the derepression of cholesterol biosynthesis by ubiquitination and destabilization of SREBP2. These results suggest a previously unknown link between ChREBP and the regulation of cholesterol synthesis with a putative role in liver injury [38]. Using tissue-specific ChREBP deletion, an essential role for intestinal (but not hepatic) ChREBP in fructose tolerance has been established [39]. The coordinated induction of glycolytic and lipogenic gene expression requires both SREBP-1c and ChREBP. Whereas SREBP-1c mediates insulin’s induction of lipogenic genes, ChREBP mediates the glucose induction of both glycolytic and lipogenic genes in an insulin-independent way. These complementary actions ensure that the liver synthesizes FAs only when insulin and carbohydrates are both present [40].

In humans, low levels of ChREBP and de novo lipogenesis in adipose tissue are associated with insulin resistance. In mice, the adipose tissue-specific knockout of ChREBP causes insulin resistance, probably due to an impairment on glucose transport and lipogenesis in this tissue [41]. In the liver, ChREBP deletion impairs hepatic insulin sensitivity and alters glucose homeostasis in mice [42]. Finally, it has been demonstrated that in brown adipose tissue (BAT), the AKT2–ChREBP pathway is induced by cold to optimize fuel storage and thermogenesis [43]. Recently provided evidence suggests that AKT2 drives de novo lipogenesis in this tissue by inducing ChREBPβ transcription. This pathway is required for optimum BAT function and is conserved in humans. These findings have important implications for understanding BAT activity under human-relevant environmental conditions.

## 3. Fat. The Peroxisome Proliferator-Activated Receptors (PPARs)

**Figure 3:**
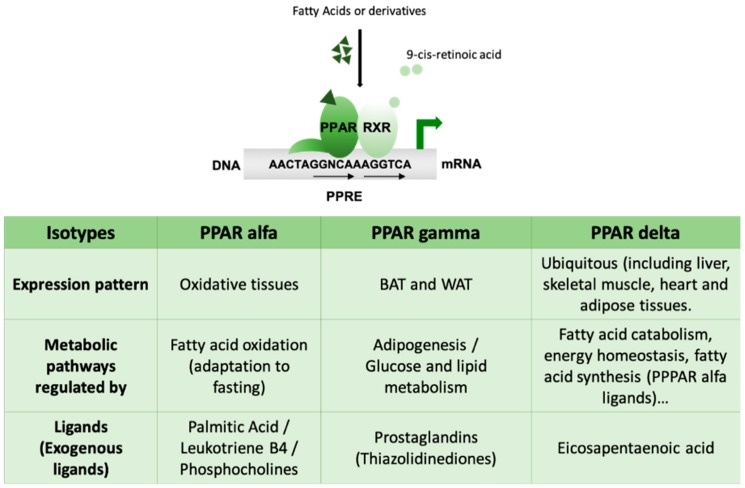
PPARs belong to the ligand-activated nuclear receptor (NR) family. They are transcription factors that exert their effects as heterodimers with the retinoid X receptor (RXR) by binding to a specific sequence of DNA called PPAR-responsive element (PPRE) with a repetitive consensus hexamer (AGGTCA). Three PPAR isotypes are described (α, β and γ) with different expression patterns and metabolic functions. PPARs are lipid sensors and can be activated by both dietary fatty acids (FAs) and their derivatives in the body and, consequently, redirect metabolism. In the liver, PPARα and PPARδ exhibit opposing activities in the control of diurnal lipid metabolism. PPARα is upregulated in the fasted state to regulate fat catabolism. By contrast, PPARδ is most active in the fed state and controls the transcription of lipogenic genes. BAT, brown adipose tissue; WAT, white adipose tissue.

Deregulation of lipid metabolism lies at the base of the most common medical disorders in western populations, such as cardiovascular disease, obesity, diabetes and fatty liver conditions. However, a gap in knowledge still exists in both the basic science and the clinical fields regarding the impact of altered lipid storage on human diseases. At the beginning of the 1960s, a diverse group of pesticides (clofibrate) were recognized as capable of causing the proliferation of peroxisomes in rat livers. Subsequently, it was identified that these compounds bound to a nuclear receptor that was known as the peroxisome proliferator-activated receptor (PPAR) (Figure 3).

The PPARs belong to the ligand-activated nuclear receptor (NR) family and the steroid receptor superfamily. The nuclear receptors are a family of transcription factors that can exert their effects as monomers, homodimers or heterodimers by binding to a specific sequence of DNA called nuclear receptor responsive elements (NRREs) with a repetitive consensus hexamer (AGGTCA) that is recognized by the DNA-binding domain (DBD) of the NR. All NRs share a common structure, a NH2 terminal region (A/B) and a conserved DBD (region C) that includes two Zn fingers, a linker region (D) responsible for nuclear localization and, finally, a well-conserved carboxy-terminal ligand-binding domain, the LBD, or region E. Some of the NR may possess an extra F domain—a highly variable carboxy-terminal tail with unknown functions, so far [44,45].

PPARs regulate the expression of genes involved in a variety of processes concerning metabolic homeostasis by controlling the metabolism of glucose and lipids, adipogenesis, insulin sensitivity, immune response, cell growth and differentiation [46]. For the PPAR-mediated transcriptional activation of its target genes, the heterodimerization of a PPAR with the RXR and the binding of the heterodimer to a PPAR responsive element (PPRE) sequence are necessary, producing a change in chromatin structure indicated by ligand activation of the complex and histone H1 release. The binding of the ligand triggers a conformational change that will generate new specific contacts with coactivators [47]. As PPARs control lipid homeostasis (lipid synthesis and oxidation) and are activated by lipids (or a closely related derivative) that act as ligands (see below), the mechanism of activation by lipids may necessarily be far more involved than the description presented here.

### 3.1. PPAR Isotypes and Metabolic Integration

Despite their different tissue distribution, this subfamily of NR functions in an integrated network to regulate metabolism. The PPARs function as lipid sensors in a way that can be activated by both dietary fatty acids (FAs) and their derivatives in the body, consequently redirecting metabolism.

The alpha isoform of the PPARs (PPARα) has a crucial role in fatty acid oxidation (FAO) and therefore is mainly expressed in highly oxidative tissues such as the liver and, to a lesser extent, in the heart, kidneys, skeletal muscle and BAT. PPARα has been shown to play a crucial role in the adaptive response to fasting by regulating genes involved in FAO [48,49] and, therefore, has indirect effects on other metabolic pathways and energy homeostasis [47,50,51].

PPARγ is highly enriched in both BAT and white adipose tissue (WAT). It is induced during adipocyte differentiation and is an important regulator of fat cells [52,53]. This member of the PPARs is a master effector of adipogenesis in a transcriptional cascade involving C/EBP [54] and has an important role in the regulation of glucose and lipid metabolism. It also participates in the regulation of cardiovascular disease, inflammation, organ development and tumor formation [55]. According to its functions, PPARγKO mice do not develop adipose tissue [56] and, in humans, a dominant negative mutation in a single allele of PPARG (encoding for PPARGγ) leads to insulin resistance and lipodystrophy phenotype [57]. Finally, this transcription factor is of great clinical importance because it is the molecular target for thiazolidinedione (TZD). TZDs are a class of antidiabetic agents that improve peripheral insulin sensitivity and assist in glycemic control in type 2 diabetic patients [58].

The third member of this family, PPARδ, has been more elusive. Its expression is quite ubiquitous and the first functions described for PPARδ were those related to the catabolism of fatty acids and energy homeostasis [50]. It is an important metabolic regulator in different tissues, such as adipose tissue, skeletal muscle and the heart [59].

The transcriptional activation of PPARδ enhances fatty acid catabolism and energy uncoupling, decreasing TG stores, improving endurance performance and enhancing cardiac contractility. Its receptor activation decreases macrophage inflammatory responses and modulates lipoprotein metabolism to lower TG while, on the other hand, raising HDL cholesterol. In liver, the activation of this transcription factor ameliorates glucose homeostasis by repressing hepatic glucose output [59].

In muscle, a fundamental role in the regulation of mitochondrial FAO is attributed to PPARδ. Thus, overexpression of PPARδ in muscle increases oxidative capacity in a marked way. In fact, mice that express large amounts of PPARδ in muscle (marathon mice) can run for hours without stopping [60]. However, in the liver, PPARδ plays a lipogenic role as indicated by overexpression (adenovirus) experiments [61] on knockout animal models [62]. Recently, it has been shown that PPARδ controls the diurnal expression of lipogenic genes in the dark/feeding cycle. Surprisingly, liver-specific PPARδ activation increases, whereas hepatocyte-PPARδ deletion reduces muscle fatty-acid uptake (see below) [63].

### 3.2. New Fats are the PPARα Endogenous Ligands

PPARα-null mice develop a phenotype characterized by hypoglycemia, hyperlipidemia, hypoketonemia and fatty liver due to their inability to meet energy demands in a fasting state [51]. FASKOL mice lack the capacity for synthesizing fatty acid from carbohydrates due to the deletion of FASN [64]. This animal, when either fed a diet without fat or exposed to prolonged fasting, has shown the same hypoglycemic phenotype as PPARα-null mice, with decreased expression of PPARα target genes. FASKOL mice have also developed a cholesterol phenotype not dependent on diet. In these cases, both hypoglycemia/steatohepatitis and cholesterol phenotypes were reversed by the administration of a PPARα agonist such as WY14643 [64]. Because the “new fat” comes from diet or from de novo synthesis via FASN, this experiment has led to the concept that only “new fat” is the capable of activating PPARα and promoting gluconeogenesis and FAO. By contrast, “old fat”, the fat mobilized from peripheral fat stores and transported to the liver where it accumulates, fails to activate PPARα. Elsewhere, by immunoprecipitation of PPARα, an endogenous ligand with nanomolar affinity was described for PPARα activation, 1-palmitoyl-2-oleoyl-sn-glycerol-3-phosphocholine (16:0/18:1 PC) [65].

Interestingly, liver PPARδ expression can generate the PPARα endogenous ligands. PPARδ overexpression (adenoviral-mediated PPARδ) up-regulates glucose utilization and de novo lipogenesis pathways [61].

Deletion of hepatocyte-PPARδ reduces, while liver-specific activation PPARδ increases, muscle fatty acid uptake [63]. Metabolite studies identify 1-stearoyl-2-oleoyl-sn-glycero-3-phosphocholine (18: 1/18: 0 PC) as a serum lipid regulated by hepatic PPARδ diurnal activity. This lipid (18: 1/18: 0 PC) increases the use of fatty acids through muscle PPARα and reduces the levels of postprandial lipids [63]. Therefore, it seems that a PPARδ-dependent signal couples the metabolism of lipids in the liver and the muscular FAO.

## 4. Amino Acids as Signaling Molecules from Restriction/Deficiency to Protein

Together with carbohydrates and lipids, proteins are the third class of macronutrients acquired through the diet. Protein intake is essential for life, mainly for acquiring essential amino acids (EAA) to maintain protein turnover and support almost all cellular processes. Protein turnover is the net result of protein synthesis and degradation and it ensures maintenance of protein functionality. The effects of amino acids and proteins on transcriptome and metabolome take place when protein turnover is unbalanced: Greater protein breakdown/less synthesis/high-protein intake leads to an increase in amino acid pools, while greater synthesis/less breakdown/low-protein intake results in a reduction in the amino acid pools [66]. The maintenance of amino acid homeostasis depends on a cell’s capacity to sense amino acid availability.

### 4.1. Amino Acid Response (AAR): The GCN2/ATF4 Pathway to Sense Low Amino Acid Levels

Higher organisms are unable to synthesize the 20 amino acids required for protein synthesis in sufficient amounts to meet cellular needs, and some of them, the EAA, must be supplied by the diet. In humans, the sources of dietary proteins are essentially animals and plants. The amount and composition of these proteins are different, and its quality depends on the content of the above-mentioned EAA. A healthy and balanced diet must cover all the requirements in amino acids and should include proteins from different sources and in different proportions.

The circulating levels of amino acids depend on the ratio between protein synthesis and protein breakdown. Besides protein turnover, aminoacidemia is directly proportional to protein intake and is strongly affected by stress situations such as trauma, thermal burning, sepsis or fever.

Amino acid response (AAR) is the canonical pathway to respond to amino acid deficiency. The reduction of EAA levels below the cell threshold causes the deacetylation of the corresponding tRNAs. These uncharged tRNAs are able to bind and activate the general control nonderepressible 2 (GCN2) kinase and to initiate the AAR signaling transduction cascade. GCN2 is considered a direct sensor of amino acids [67]. When activated, GCN2 phosphorylates the eukaryotic initiation factor 2 alpha (eIF2α) [68,69], which results in the activation of the integrated stress response (ISR) to maintain cellular homeostasis [70]. ISR activation reduces general protein synthesis by the slowing or stalling of the initiation step of mRNA translation through a downregulation of the eIF2B activity [71]. Paradoxically, in this situation there is an increase in the translation of discrete mRNAs including the activating transcription factor 4 (ATF4) [72,73]. Once induced, ATF4 directly or indirectly triggers the transcription of a subset of specific target genes to adapt to dietary stress [74].

**Figure 4:**
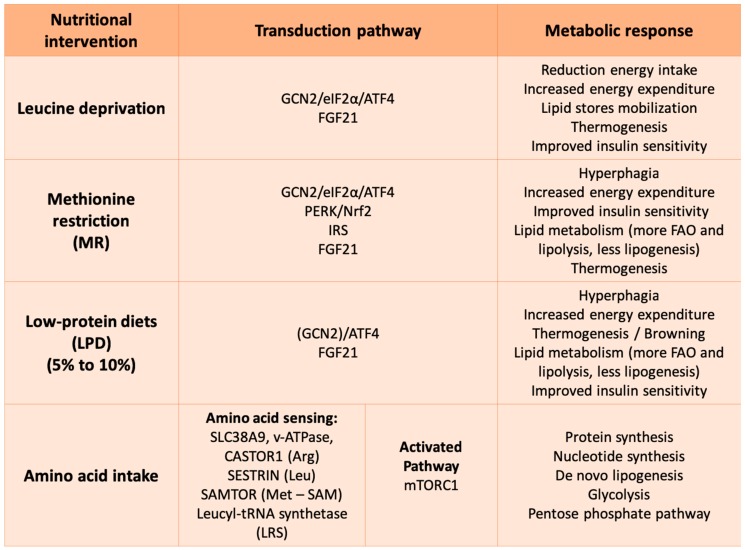
Protein intake is essential for acquiring essential amino acids (EAA) to maintain protein turnover and support almost all cellular processes. The effects of amino acids and proteins on transcriptome and metabolome take place when the protein turnover is unbalanced and there are changes in the amino acid pool. Amino acid-restricted diets, LPD and protein intake have an impact on metabolic homeostasis and directly affect not just protein metabolism but also lipid and glucose metabolism.

Although the GCN2/eIF2α/ATF4 is the major signaling pathway to respond to amino acid starvation, it is not unique [75]. It has been reported that a methionine-restricted (MR) diet activates a noncanonical protein kinase R-like endoplasmic reticulum (ER) kinase (PERK)/nuclear factor-like 2 (Nrf2) axis [76]. Along the same lines, Laeger et al. demonstrated that the absence of GCN2 is compensated upstream of ATF4 to maintain an increased expression of FGF21 in long-term protein-restricted diets [77]. Finally, at least in part, the activation of the IRS signaling pathway in the liver under an MR diet seems to be independent of p-eIF2 [78] (Figure 4).

### 4.2. Metabolic Impact of Amino Acid Restricted/Deprived Diets

Besides protein homeostasis, the dietary content of amino acids has a direct impact on lipid metabolism [79,80], health and lifespan. Leucine-deprived mice have shown a reduction in energy intake, increased energy expenditure (EE) and mobilization of the lipid stores [81] through transcriptional effects on the liver, WAT and BAT. In these animals, there was an increment of sympathetic outflow to adipose tissues, an induction in the expression of FAO genes linked to a reduction in the expression of lipogenic genes and FASN activity in WAT and an overexpression of uncoupling protein 1 (UCP1) and type 2 deiodinase (Dio2) in BAT [82,83]. In the liver, a leucine-deprived diet produces decreases in genes associated with fatty acid and TG synthesis, but not in genes linked to fatty acid transport or oxidation [81]. It has been described that the decrease in expression of SREBP-1c in the liver and WAT is the responsible mechanism for a reduction in the expression of lipogenic genes in a leucine-deprived diet [84].

All these effects cause weight loss, a reduction of fat mass and an improvement in insulin sensitivity, probably through the activation of the AMP-activated protein kinase and a GCN2-dependent decrease in the mammalian target of rapamycin (mTOR)/S6 kinase 1 (S6K1) signaling [68,85].

In the same way, MR diets show similar effects on lipid metabolism [86,87,88,89], insulin sensitivity [90] and mitochondrial uncoupling [87]. The metabolic response to MR diets administered to rats and mice includes hyperphagia, increased EE, improvement in insulin sensitivity and reduced fat deposition, liver TGs and circulating lipids [86,91,92], besides changes in membrane phospholipid composition [93]. In mice, WAT responds to MR by increasing the expression of genes involved in FAO and the upregulation of FASN and SCD1 in WAT, but also by the downregulation of lipogenic genes in the liver [92]. This liver reduction of lipid content has also been observed in patients with metabolic syndrome [94]. Finally, EAA deprivation changes the levels of anorexigenic neuropeptides and their signaling in hypothalamic feeding centers [95,96,97].

The metabolic response to amino acid starvation or amino acid-deficient diets has been linked to FGF21. The changes described in lipid metabolism in the liver, WAT and BAT are impaired in FGF21-deficient mice [98,99,100]. FGF21 is a member of the Fibroblast Growth Factor (FGF) family, which is mainly produced by the liver but also by other tissues such as WAT and BAT, skeletal muscle and pancreatic beta cells [101,102]. Its expression is regulated among other transcription factors by ATF4 [103], pointing out the GCN2/eIF2α/ATF4 as the major pathway to induce FGF21 expression by low-protein diets (LPD) or leucine-deprived diets [103].

Animals fed an MR diet are resistant to diet-induced obesity, showing improved glucose homeostasis, increased FA activation and oxidation in the liver, increased lipolysis in WAT, increased Ucp1 expression in BAT [90,104,105] and increased circulating levels of FGF21. FGF21 induction under MR diets has also been described by several authors, and it has been demonstrated that FGF21 is a critical mediator of the metabolic effects of an MR diet on EE, WAT remodeling and insulin sensitivity, but not on hepatic gene expression [106]. Moreover, Wanders et al. described that the overexpression of FGF21 in an MR diet is independent of GCN2 signaling [76]. Regarding methionine, some authors point out cysteine as the key player on the metabolic effects of MR diets, and have described how cysteine supplementation attenuates the metabolic response to an MR diet [107,108].

Finally, it should be noted that not just EAA-deficient/deprived diets exert effects on metabolism. Although some differences have been described between protein-free (0% protein calories), very-low-protein (5% protein) and moderately low-protein (10% protein) diets [109] regarding food intake and EE induction, globally, LPDs have shown comparable metabolic phenotypes to leucine or methionine restriction [110]. LPD causes weight loss and an increase in both food intake and EE [110,111]. In both rodents and humans, LPD induces FGF21 circulating levels [111,112] and thermogenic markers in the BAT of obese rats [109]. In line with a leucine-deprived diet, the effects on lipid metabolism, food intake and EE observed in LPD are blunted in FGF21 liver-specific knockout mice (L*Fgf21*KO), showing that FGF21 is involved in the metabolic response to protein-restricted diets [108,110].

The impairment of the GNC2 signaling pathway has dramatic consequences in animals fed amino acid restricted diets [68,81]. GCN2 knockout mice have shown hepatic steatosis and reduced muscle mass under a leucine-deprived diet. Moreover, a double-knockout mouse with a genetic deletion of GCN2 and the branched chain keto acid dehydrogenase kinase (BDK) will die in less than two weeks postnatal [113]. These effects are not present when animals are provided with enough EAA. Under a normal diet, rodents have not shown any metabolic phenotype. These data indicate that defects on GCN2 are revealed only when challenged with amino acid deficiency. In humans this could be important for the design of personalized nutritional therapies.

### 4.3. mTOR Signaling Pathway to Sense Amino Acid Availability

The mTOR is a serine/threonine kinase ubiquitously expressed. In humans, mTOR is the core protein of two different multiprotein complexes, TORC1 and TORC2. Of the two complexes, TORC1 is the one that integrates nutritional signals, the energy status of the cells and their stress levels [114,115]. TORC1 is activated by growth factors but also when enough energy, oxygen and building blocks such as amino acids are present; it is inhibited during stress or fasting, when a lack of resources prevents the turning on of the anabolic pathways [116] (Figure 4).

The activation of TORC1 by amino acids occurs in most cases through the RAG GTPase complex [117,118]. This RAG complex is located in the membrane of the lysosomes associated with the RAGULATOR complex, a pentameric complex [119,120]. The presence of amino acids triggers the conversion of the RAG proteins into their GTP-bound state, which enables them to recruit TORC1 to the lysosome via an interaction with the RAPTOR subunit of the TORC1 complex. Besides its interaction with RAG, TORC1—through the catalytic domain of mTOR—also interacts in the lysosome with the protein RHEB (RAS homolog enriched in the brain), responsible for the TORC1 activation by growth factors [121,122]. Because RHEB depletion blocks the amino acid-dependent activation of TORC1, it has been postulated that full activation of TORC1 requires growth factors and amino acids [118,120].

The identification of amino acids’ cellular sensors, and the way they activate TORC1, are far from the final map. It is known that TORC1 senses cytosolic and intralysosomal amino acids. Some recent studies have described the lysosomal arginine sensor SLC38A9 as necessary for the efflux of EAA and the activation of TORC1 [123,124,125,126]. It has also been demonstrated that SLC38A9 interacts with a v-ATPase that is associated with the RAGULATOR complex and acts as an activator of the RAG complex [123,124,125,126,127]. Moreover, it has been published that a key role of the v-ATPase is signaling the lysosomal amino acids, but nothing is known about how this ATPase can sense intralysosomal amino acids.

Different mechanisms have been postulated to sense cytosolic amino acids. The protein complex GATOR1/GATOR2 regulates TORC1 activity and is the main pathway to sense amino acids [128,129,130].

GATOR1 is linked to the lysosomal membranes by the KICSTOR complex and inhibits TORC1 through its GTPase-activating protein (GAP) activity toward RAG. On the other hand, GATOR2 is able to block the GAP activity of GATOR1 [128], thus activating TORC1. The question is how GATOR proteins are regulated by amino acids. CASTOR1, SESTRIN and SAMTOR have been identified as cytosolic amino acid sensors for TORC1 activation. CASTOR1 is an arginine sensor that binds and blocks GATOR2 when arginine is absent. The binding of arginine to CASTOR1 blocks its interaction with GATOR2 and causes the activation of TORC1 [131,132]. A similar mechanism has been proposed for SESTRIN, which senses leucine levels. In this case, leucine prevents the interaction between SESTRIN and GATOR2, also triggering the activation of TORC1 [132,133,134,135]. Finally, SAMTOR is a methionine sensor that detects *S*-adenosylmethionine (SAM). SAMTOR can bind directly to GATOR1 when levels of SAM are high. In a methionine-starvation situation, levels of SAM decrease and the SAMTOR–GATOR1 interaction is disrupted leading to a reduction in TORC1 activity [136].

Although most of the amino acids are sensed by the GATOR1/GATOR2 complex, some alternative pathways have been described. Glutamine, for instance, is sensed via the RAG-related ARF family GTPases [137]. The FLCN complex has GAP activity toward RAG and is activated by amino acids, thus activating TORC1 signaling [138,139].

Finally, the leucyl-tRNA synthetase (LRS) has also been postulated as an amino acid sensor able to regulate TORC1 activity. Some authors have proposed that LRS could interact directly with RAG and act as a GAP [138,140] but others have shown that LRS leucylates a lysine residue of RAG and activates TORC1 [141].

### 4.4. Metabolic Impact of TORC1 Activation: Protein Synthesis, de novo Lipogenesis, Glycolysis and Pentose Phosphate Pathway

The TORC1 complex controls cell growth by promoting protein and lipid synthesis, cell cycle, and anabolic pathways and blocking catabolism and autophagy. This section is focused on the impact of TORC1 activity on protein, lipids and glucose metabolism.

TORC1 phosphorylates the p70S6 Kinase 1 (S6K1) and the eukaryotic translation initiation factor 4E (eIF4E) binding protein (4EBP) [116].

S6K1 is a serine/threonine protein kinase that, when activated, phosphorylates several proteins related to the initiation step of the mRNA translation [142]. S6K1 activates the eukaryotic initiation factor 4B (eIF4B), which belongs to the 5’ cap-binding eIF4F complex where it acts as a positive regulator. On the other hand, S6K1 phosphorylates and triggers proteasomal degradation of the eIF4B-inhibitor PDCD4 [143].

The 4EBP is phosphorylated by TORC1 and this causes its dissociation from the protein eIF4E. In its dephosphorylated form, 4EBP blocks the protein translation by binding to the eIF4E and preventing the assembly of the eIF4F complex [144,145].

TORC1 promotes de novo lipogenesis through the activation of SREBP1. The activation of SREBP under TORC1 signaling takes places through two different mechanisms. The first depends on S6K1 activity that, via an unknown molecular mechanism, is able to promote the processing of SREBP1 [146,147,148]. The second mechanism involves the phosphorylation of LIPIN1 by TORC1. TORC1 phosphorylates and controls the entry of LIPIN1 to the nucleus. When dephosphorylated, LIPIN1 is active and inhibits SREBP transcriptional activity. Once phosphorylated by TORC1, LIPIN1 cannot enter the nucleus and SREBP is active [149]. Both mechanisms increase the gene expression of enzymes involved in cholesterol and lipid biosynthesis

Regarding glucose metabolism, TORC1 increases HIF1a protein levels by inducing its translation. HIF1a promotes the gene expression of glycolytic enzymes and glucose uptake. The induction of glycolysis and the reduction of oxidative phosphorylation downstream of TORC1 signaling facilitates the incorporation of nutrients as biosynthetic precursors instead of energy suppliers. Finally, the activation of SREBP by TORC1 also promotes the gene expression of enzymes from the oxidative arm of the pentose phosphate pathway that will generate NADPH for biosynthesis [146].

It is described that the impairment of mTORC1 signaling drives the development of cancer, obesity and cardiovascular disease.

## 5. Concluding Remarks

In this review, we have summarized the molecular mechanisms of diet-induced gene expression, which allows the integration of nutrient signaling to metabolic homeostasis. Although not discussed in this paper, it is well-known that dysregulations on the above-mentioned signaling transduction pathways trigger the development and progression of metabolic disorders such as obesity and type 2 diabetes, thus revealing a complicated network of regulatory mechanisms to achieve metabolic homeostasis.

The connection between alterations in the signaling pathways and metabolic diseases is particularly well-illustrated in the case of PPARγ. Mutations in the gene coding for PPARγ are clearly related to an obese phenotype and insulin resistance in humans. Thiazolidinediones (TZDs) are efficacious therapeutic agents for the treatment of noninsulin-dependent diabetes. These drugs improve insulin sensitivity through the modulation of glucose and fatty acid metabolism, are high-affinity ligands for PPARγ and their antidiabetic activity is mediated through the activation of this nuclear receptor.

This example points out the importance of the knowledge/understanding of molecular mechanisms that through regulating gene expression control metabolism in response to dietary inputs to design new therapeutic strategies against metabolic diseases based on nutritional interventions.

## Abbreviations

AAR	Amino acid response
ATF4	Activating transcription factor 4
BAT	Brown adipose tissue
ChoRE	Carbohydrate response element
ChREBP	Carbohydrate responsive element binding protein
DBD	DNA binding domain
Dio2	Type 2 deiodinase
EAA	Essential amino acids
EE	Energy expenditure
eIF2α	Eukaryotic initiation factor 2 alpha
eIF4B	Eukaryotic initiation factor 4B
FAO	Fatty acid oxidation
FASN	Fatty acid synthase
FGF21	Fibroblast growth factor 21
FXR	Farnesoid X receptor
G6P	Glucose 6-phosphate
GAP	GTPase activating protein
GCN2	general control nonderepressible 2
GK	Glucokinase
HBP	Hexosamine biosynthetic pathway
ISR	Integrated stress response
LBD	Ligand binding domain
LPD	Low protein diet
LRS	Leucyl-tRNA synthetase
LXR	Liver X Receptor
MR	Methionine-restricted
mTOR	Mammalian target of rapamycin
NR	Nuclear receptors
NRF2	nuclear factor-like 2
NRRE	Nuclear receptors responsive element
O-GlcNAc	O-linked N acetylglucosamine
PERK	protein kinase R-like endoplasmic reticulum (ER) kinase
PPAR	Peroxisome proliferator activated receptor
PPRE	PPAR responsive element
RHEB	Ras homolog enriched in the brain
S6K1	S6 kinase 1
SAM	S-adenosylmethionine
SCD1	Stearoyl-CoA desaturase
SREBP	Sterol regulatory element binding protein
TZD	Thiazolidinediones
UCP1	Uncoupling protein 1
WAT	White adipose tissue
X5P	Xylulose 5-phosphate
